# *Matrix metalloproteinase-9 *gene polymorphisms in nasal polyposis

**DOI:** 10.1186/1471-2350-11-85

**Published:** 2010-06-09

**Authors:** Ling-Feng Wang, Chen-Yu Chien, Chih-Feng Tai, Wen-Rei Kuo, Edward Hsi, Suh-Hang Hank Juo

**Affiliations:** 1 Department of Otolaryngology, College of Medicine, Kaohsiung Medical University, Kaohsiung, Taiwan; 2 Graduate Institute of Medicine, Kaohsiung Medical University, Kaohsiung, Taiwan; 3 Department of Medical Genetics, College of Medicine, Kaohsiung Medical University, Kaohsiung, Taiwan; 4 Department of Otolaryngology, Kaohsiung Municipal Hsiao-Kang Hospital, Kaohsiung Medical University, Kaohsiung, Taiwan; 5 Department of Otolaryngology, Kaohsiung Medical University Hospital, Kaohsiung, Taiwan; 6 Department of Medical Research, Kaohsiung Medical University Hospital, Kaohsiung, Taiwan

## Abstract

**Background:**

Matrix metalloproteinase (MMP) is involved in the upper airway remodeling process. We hypothesized that genetic variants of the *MMP-9 *gene are associated with cases of chronic rhinosinusitis with nasal polyposis.

**Methods:**

We conducted a case-control study where 203 cases of chronic rhinosinusitis with nasal polyposis and 730 controls were enrolled. Three tagging single nucleotide polymorphisms (SNPs) and one promoter functional SNP rs3918242 were selected. Hardy-Weinberg equilibrium (HWE) was tested for each SNP, and genetic effects were evaluated according to three inheritance modes. Haplotype analysis was also performed. Permutation was used to adjust for multiple testing.

**Results:**

All four SNPs were in HWE. The T allele of promoter SNP rs3918242 was associated with chronic rhinosinusitis with nasal polyposis under the dominant (nominal p = 0.023, empirical p = 0.022, OR = 1.62) and additive models (nominal p= 0.012, empirical p = 0.011, OR = 1.60). The A allele of rs2274756 has a nominal p value of 0.034 under the dominant model and 0.020 under the additive model. Haplotype analysis including the four SNPs showed a global p value of 0.015 and the most significant haplotype had a p value of 0.0045. We did not see any SNP that was more significant in the recurrent cases.

**Conclusions:**

We concluded that *MMP-9 *gene polymorphisms may influence susceptibility to the development of chronic rhinosinusitis with nasal polyposis in Chinese population.

## Background

Tissue remodeling has gained increasing interest in the pathogenesis of chronic upper and lower airway disease. It is a dynamic process that involves extracellular matrix (ECM) production and degradation leading to either a normal reconstruction process or a pathological structure [1]. Nasal polyposis is a chronic inflammatory disease in the upper airway. The histological appearance of nasal polyposis is characterized by inflammatory cell infiltration, modifications of epithelial cell differentiation, and tissue remodeling that includes basement membrane thickening, gland modifications, ECM accumulation and edema. Previous studies suggested that polyposis formation involved ECM protrusion through an initial localized epithelial defect [2]. Interactions between epithelial, stromal, and inflammatory cells could then perpetuate further polyposis growth. Although inflammatory cells, especially eosinophils, are thought to play an important role in nasal polyposis, the sequence of events for the development of polyposis is still controversial.

Matrix metalloproteinases (MMPs) are a family of zinc and calcium-dependent endopeptidases that are known to be important to remodel the ECM. *MMP-9 *(92 kD type IV Collagenase, gelatinase B) cleaves type IV collagen which is the major structural component of basement membrane. Studies also suggested that *MMP-9 *may play a crucial role in airway remodeling in asthma [3]. In transgenic animal studies, an elevated level of *MMP-9 *was associated with defects in bronchial architecture [4]. Upregulation of *MMP-9 *in the nasal polyposis may damage the collagen of basement membrane of epithelia and blood vessels, causing increases in permeability and edema in the stroma. Elevated levels of *MMP-9 *protein [5-8] and *MMP-9 *mRNA [6,7] were found in the cases of nasal polyposis. Elevated plasma *MMP-9 *level was also reported recently in patients with allergic nasal polyps [9]. Patients with poor-healing reaction after sinus surgery had more severe edematous and fibrotic changes [10], and higher amounts of *MMP-9 *in both nasal secretions [11] and connective tissue [12] when compared with good healers. Therefore *MMP-9 *may play a role in the pathogenesis or recurrence of the nasal polyposis. To our knowledge, there is no published data regarding the relationship between *MMP-9 *genetic polymorphisms and the risk of the chronic rhinosinusitis with nasal polyposis. We conducted a case-control study to systematically investigate the role of *MMP-9 *tagging single nucleotide polymorphisms (tSNPs) and a promoter functional polymorphism in the development of chronic rhinosinusitis with nasal polyposis in a Chinese population residing in Taiwan.

## Methods

### Subjects

We recruited 203 patients of bilateral chronic rhinosinusitis with nasal polyposis at the Kaohsiung Medical University Hospital between October 2005 and June 2007. The diagnosis of chronic rhinosinusitis with nasal polyposis was based on history, physical examination, nasal endoscope, and sinus CT scan. Patients with malignancies or asthma were excluded from the study. All the patients were followed up at least three months after surgery. Recurrence was defined as a patient with newly developed pedunculated nasal polyps (instead of cobblestone or polypoid mucosa) fully occupying the middle meatus three months after surgery using either anterior rhinoscope or nasal endoscope. Recurrence was identified in twenty six patients for whom revision surgery was performed at our institution. A total of 730 control subjects were used in the present study and they were recruited from the general populations who volunteered to participate in our study while receiving a health screening examination at our Hospital. None of the controls reported major disable diseases upon enrollment. Information on demographic characteristics was collected. The study was proved by the Institutional Review Board (IRB) of Kaohsiung Medical University Hospital and written informed consent was given by each subject.

### SNP selection and Genotyping

Genomic DNA was extracted from peripheral blood by a standard method. Three tSNPs were selected from the HapMap Project (International HapMap Consortium) and all of them have the minor allele frequency ≥ 10% in the Han Chinese population. The three tSNPs are: the non-synonymous SNP rs2664538 (Gln/Arg) at exon 6 (this SNP was merged to rs17576), intronic SNP rs3787268 at intron 8 and non-synonymous SNP rs2274756 (Gln/Arg) at exon 12 (this SNP was merged to rs17577). In addition, we also chose one commonly studied promoter functional SNP (rs3918242, i.e. -1562 C/T). Genotyping for the three tSNPs was carried out by using the TaqMan 5' nuclease assay (Applied Biosystems, Foster City, USA). Briefly, PCR primers and TaqMan Minor Groove Binder (MGB) probes were designed and reactions were performed in 96-well microplates with ABI 9700 thermal cyclers (Applied Biosystems, Foster City, USA). Fluorescence was measured with an ABI 7500 Real Time PCR System (Applied Biosystems, Foster City, USA) and analyzed with its System SDS software version 1.2.3. Genotyping for promoter SNP rs3918242 (-1562C/T) was determined by the polymerase chain reaction (PCR)-restriction fragment length polymorphism (RFLP) method. The forward and reverse primers were 5' GCCTGGCACATAGTAGGCCC 3' and 5' CTTCCTAGCCAGCCGGCATC 3', respectively [13]. The restriction site was detected by SphI [13].

### Statistical analysis

Continuous variables were analyzed by independent t test and were presented as mean ± SD. The allele frequency was obtained by direct gene counting. Hardy-Weinberg equilibrium (HWE) was checked in controls by using the x^2 ^test. Multiple logistic regression analysis was performed to assess the genetic effect with adjustment for age and sex. We examined the effect of the minor allele of each SNP in three genetic models (dominant, additive, and recessive). We also performed a subset analysis by stratifying the cases according to recurrence of the disease. SPSS 13.0 version for Windows (Chicago, IL, USA) was used for statistical analysis. Linkage disequilibrium (LD) was assessed for any pair of SNPs and haplotype blocks were defined using the default setting of the Haploview software [14]. We used the Hap-Clustering program [15] to evaluate haplotype-phenotype association. To adjust for multiple testing, we also presented empirical p values by 10,000 permutations.

## Results

### Study Participants

Table 1 shows the baseline characteristics of the subjects. The mean age (years) was 43.8 ± 16.5 in cases and 54.4 ± 11.9 in controls. The sex distribution in cases was 2.1:1 (male to female) which is similar to the ratio reported previously [16,17], and was 1:1.4 in controls. Sixty-four patients (31.5%) had recurrent nasal polyps.

**Table 1:** Baseline demographics of study characteristics

Characteristic	Cases (n = 203)	Control (n = 730)
Age, mean (SD)	43.8 (16.5)	54.4 (11.9)
Sex (male to female ratio)	137/66 (2.1:1)	299/420 (1:1.4)
Smoker	39 (19.2%)	130 (17.8%)
Nasal polyposis recurrence, No.(%)		
Yes	64 (31.5%)	
No	139 (68.5%)	

### Single SNP Results

The distribution of *MMP-9 *genotypes was in HWE among controls (all p values > 0.05) for all SNPs. The genotyping call rates ranged from 92% to 98%. In the analysis of overall data (Table 2), the multivariate logistic regression model showed that the T allele of promoter SNP rs3918242 was associated with chronic rhinosinusitis with nasal polyposis under the dominant (nominal p = 0.023, empirical p = 0.022, OR = 1.62) and additive models (nominal p = 0.012, empirical p = 0.011, OR = 1.60). The A allele of rs2274756 had a nominal p value of 0.034 under the dominant model and 0.020 under the additive model. The r^2 ^between these two significant SNPs (rs3918242 and rs2274756) was 0.727. The exonic SNP rs2274756 became insignificant when the regression model also included the promoter SNP rs3918242. We did not find any significant results for SNP rs3787268 and rs2664538 in any of the three genetic models. In the subset analysis, none of the SNPs in the recurrent patients had a significant P value, except for the A allele of rs2664538 under a recessive model. The p values in the non-recurrent cases showed a similar pattern as the overall cases (Table 2).

**Table 2:** Four SNPs and their relationships with nasal polyposis under three genetic models

SNP	disease status	Genotype (n)(%)			p		
					
					Dominant	Additive	Recessive
rs3918242	case	TT(6)(3%)	CT(44)(21.7%)	CC(137)(67.5%)	0.023*	0.012*	0.097
(promoter)	control	TT(4)(0.5%)	CT(125)(17.1%)	CC(560)(76.7%)			
rs2664538	case	AA(16)(7.9%)	AG(57)(28.1%)	GG(119)(58.6%)	0.640	0.752	0.073
(in exon 6)	control	AA(37)(4.7%)	AG(246)(33.7%)	GG(428)(58.6%)			
rs3787268	case	AA(28)(13.8%)	AG(90)(44.3%)	GG(74)(36.5%)	0.503	0.345	0.361
(intron 8)	control	AA(135)(18.5%)	AG(335)(45.9%)	GG(246)(33.7%)			
rs2274756	case	AA(6)(3%)	AG(49)(24.1%)	GG(137)(67.5%)	0.034*	0.020*	0.134
(exon 12)	control	AA(5)(0.7%)	AG(147)(20.1%)	GG(572)(78.4%)			
Recurrent cases(n = 64)							
rs3918242	case	TT(2)(3%)	CT(12)(18.8%)	CC(43)(67.2%)	0.382	0.243	0.175
	control	TT(4)(0.5%)	CT(125)(17.1%)	CC(560)(76.7%)			
rs2664538	case	AA(7)(10.9%)	AG(18)(28.1%)	GG(36)(56.3%)	0.841	0.314	0.033*
	control	AA(37)(4.7%)	AG(246)(33.7%)	GG(428)(58.6%)			
rs3787268	case	AA(8)(12.5%)	AG(27)(42.2%)	GG(26)(40.6)	0.439	0.290	0.318
	control	AA(135)(18.5%)	AG(335)(45.9%)	GG(246)(33.7%)			
rs2274756	case	AA(2)(3%)	AG(15)(23.4%)	GG(44)(68.8%)	0.383	0.273	0.264
	control	AA(5)(0.7%)	AG(147)(20.1%)	GG(572)(78.4%)			
Non-recurrent cases(n = 139)							
rs3918242	case	TT(4)(2.9%)	CT(32)(23%)	CC(94)(67.6%)	0.021*	0.014*	0.159
	control	TT(4)(0.5%)	CT(125)(17.1%)	CC(560)(76.7%)			
rs2664538	case	AA(9)(6.5%)	AG(39)(28.1%)	GG(83)(59.7%)	0.356	0.592	0.561
	control	AA(37)(4.7%)	AG(246)(33.7%)	GG(428)(58.6%)			
rs3787268	case	AA(20)(14.4%)	AG(63)(45.3%)	GG(48)(34.5%)	0.990	0.824	0.690
	control	AA(135)(18.5%)	AG(335)(45.9%)	GG(246)(33.7%)			
rs2274756	case	AA(4)(2.9%)	AG(34)(24.5%)	GG(93)(66.9%)	0.037*	0.026*	0.203
	control	AA(5)(0.7%)	AG(147)(20.1%)	GG(572)(78.4%)			

Dominant, additive or recessive model: the rare allele had a dominant, additive or recessive effect

All p values were adjusted by sex and age

* p < 0.05

### LD and Haplotype Analysis

**Figure 1:**
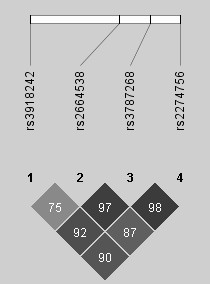
**LD plot of four *matrix metalloproteinase-9 *(*MMP9*) single-nucleotide polymorphisms**. The pairwise D' value (percentage) is displayed in each cell. The cells in dark gray indicate strong linkage disequilibrium. The bar on the top is the *MMP9 *gene structure and location of each single-nucleotide polymorphism.

The four SNPs formed one haplotype block (Figure 1). Haplotype analysis demonstrated a global p value of 0.015 (Table 3). The most significant haplotype TGGA (rs3918242/rs2664538/rs3787268/rs2274756) had the frequency of 14.7% in cases and 8.5% in controls (haplotype specific p value of 0.0045).

**Table 3:** The result from haplotype analysis which yielded a global p value of 0.015

Haplotype	Patients with chronic rhinosinusitis	Healthy controls	Total	p value
CGGG	23.0%	24.7%	24.4%	0.2532
CGGA	1.1%	2.2%	2.0%	0.0393
CGAG	38.1%	42.0%	40.9%	0.3813
CAGG	23.2%	22.5%	22.7%	0.7180
TGGA	14.7%	8.5%	10.1%	0.0045

## Discussion

We systematically investigated four SNPs at *MMP-9 *in relation to chronic rhinosinusitis with nasal polyposis in a Chinese population residing in Taiwan. The results showed that three SNPs were associated with the development of chronic rhinosinusitis with nasal polyposis. The most significant result was from the promoter polymorphism of the *MMP-9 *gene (rs3918242, i.e. -1562 C/T), which indicates that the rare T allele would significantly increase the risk for nasal polyposis. We did not find more significant results in the recurrent subjects. As a matter of fact, the frequencies of the risk T allele at rs3918242 and the risk A allele at rs2274756 are similar between the non-recurrent cases and recurrent cases. Using the haplotype analysis, the result gave us a stronger statistical support for *MMP-9 *as a genetic marker. Therefore, the current data showed that *MMP-9 *polymorphisms might influence susceptibility to the development of chronic rhinosinusitis with nasal polyposis but might not increase the risk for recurrence. To our knowledge, this is the first report to demonstrate the potential contribution of the *MMP-9 *genetic variants to the development of chronic rhinosinusitis with nasal polyposis.

Regulation of MMPs is complex. It is considered that regulation of MMP activity occurs at three levels: gene transcription, activation of the secreted proenzyme and inhibition by specific and non-specific inhibitors [18]. The regression analysis suggested that the two significant SNPs were not independent and the promoter SNP remained significant after including the rs2274756 at exon 12 into the model. Therefore, it is likely that the genetic effect of *MMP-9 *was mainly from the promoter SNP. Furthermore, *MMP-9 *protein[5-8] and mRNA[6,7] levels were reported to be different between patients with the chronic rhinosinusitis with nasal polyposis and controls. Zhang et al. [19] also reported that the T allele of promoter SNP rs3918242 at *MMP-9 *had a higher promoter activity which would lead to a higher production of *MMP-9*, although a recent study did not find any differential expression between the T and C alleles [20].Taken together, the studies using different approaches consistently imply that the high activity T allele is likely to increase a risk for chronic rhinosinusitis with nasal polyposis.

Our study design has limitations. The sample size in the present study was moderate, and type I error is always possible. However, based on the result of functional SNP rs3918242, the study population still provided a power of 85% to detect the risk allelic effect. Further studies to replicate our results are necessary. The follow-up period is limited in our study. Because nasal polyps may recur several years after surgery, extended follow up is required prior do drawing further conclusions. Our controls did not receive a nasal examination to exclude the possibility of nasal polyposis. Because the prevalence of nasal polyps is usually less than 4% [21,22], we did not expect a significant number of our controls to have chronic rhinosinusitis with nasal polyposis. Furthermore, if any controls had chronic rhinosinusitis with nasal polyposis, the effect of such misclassification of disease status would only decrease the statistical power and reduce the significance. Therefore, our conclusion is valid even though we did not examine the controls. Although the sex distribution was different between cases and controls, the genotype distributions between male controls and female controls are very similar. Accordingly, our results were not confounded by the sex effect.

## Conclusions

In conclusion, our study provides novel evidence to support genetic polymorphisms in the MMP9 gene can influence the risk for chronic rhinosinusitis with nasal polyposis. Furthermore, the T allele of the functional promoter SNP rs3918242 that has been shown to increase MMP-9 expression is also a risk allele for chronic rhinosinusitis with nasal polyposis. However, the role of MMP-9 polymorphisms in the recurrence of the disease requires further investigation.

## Competing interests

The authors declare that they have no competing interests.

## Authors' contributions

LFW performed the clinical examination of patients and participated in the coordination of the study, drafted the manuscript and participated in the design of the study. CYC carried out genotyping and performed statistical analysis. CFT and WRK participated in study design and helped drafting the manuscript. EH participated in the statistical analysis. SHHJ conceived of the study, and participated in its design and coordination and helped to draft the manuscript. All authors read and approved the final manuscript.

## Pre-publication history

The pre-publication history for this paper can be accessed here:

http://www.biomedcentral.com/1471-2350/11/85/prepub
